# Diagnosis and treatment of pure arterial malformation: Three case reports and literature review

**DOI:** 10.1097/MD.0000000000020229

**Published:** 2020-05-22

**Authors:** Tian-Yi Liu, Ning Xu, Zheng Wan, Zhe-Ming Zhang, Jian-Jun Xu, Hao Meng, Hong-lei Wang

**Affiliations:** Department of Neurosurgery, the First Hospital of Jilin University, Changchun, China.

**Keywords:** aneurysm, arterial malformation, vascular disorders

## Abstract

**Rationale:**

The incidence of pure arterial malformations is relatively low, and few cases have been reported. Only 2 cases with pure arterial malformation have been reported to receive surgery or endovascular treatment.

**Patient concerns:**

We report 3 cases and review the relevant literatures. The head examinations of the patients suggested the presence of high-density shadows in front of the pons and midbrain, the dilation of the supraclinoid segment of the right internal carotid artery, and moyamoya in the left brain with an aneurysm-like expansion located on the left posterior communicating artery respectively. After admission, head digital subtraction angiography (DSA) was performed.

**Diagnoses:**

Digital subtraction angiography (DSA) for these 3 patients showed that the left posterior communicating artery, the supraclinoid segment of the right internal carotid artery, and the left posterior communicating artery appeared dilated, tortuous, and spirally elongated. In addition, the lesions in the latter 2 patients were accompanied with local aneurysmal changes.

**Interventions:**

Two patients were given conservative treatment, and another patient was given endovascular treatment. A head DSA was reviewed 6 months after therapy.

**Outcomes:**

The prognosis status of the 3 patients was good. Two patients in the conservative treatment group showed no changes in the lesions on head DSA examination. The DSA examination of the third patient indicated that the vascular remodeling of the diseased vessels was good, the blood vessels were unobstructed, and the aneurysms had disappeared.

**Lessons:**

Pure arterial malformations mostly occur in young women and may involve any blood vessels in the brain. It can be accompanied with local aneurysms and calcification. The patients are often given conservative treatment but need to be reviewed regularly. However, it is beneficial to give endovascular treatment to the patients with local aneurysms.

## 1 Introduction

The incidence of pure arterial malformations is low; only few cases have been reported. In most patients, these are incidentally discovered during head computerized tomography (CT) or computed tomography angiography (CTA) examination performed for headache or other reasons. The concept of pure arterial malformation was first reported by McLaughlin et al, whose definition involves the presence of dilated, overlapping, and tortuous arteries forming a mass of arterial loops with a coil-like appearance in the absence of any venous components.^[1]^ Due to its rarity, the optimal treatment remains controversial. Brinjikji et al proposed that patients with pure arterial malformations should receive conservative treatment.^[2]^ In this study, we reported 3 cases of pure arterial malformation, with 1 treated with endovascular intervention, and reviewed the related literatures.

## 2 Case presentation

### 2.1 Case 1

**Figure 1:**
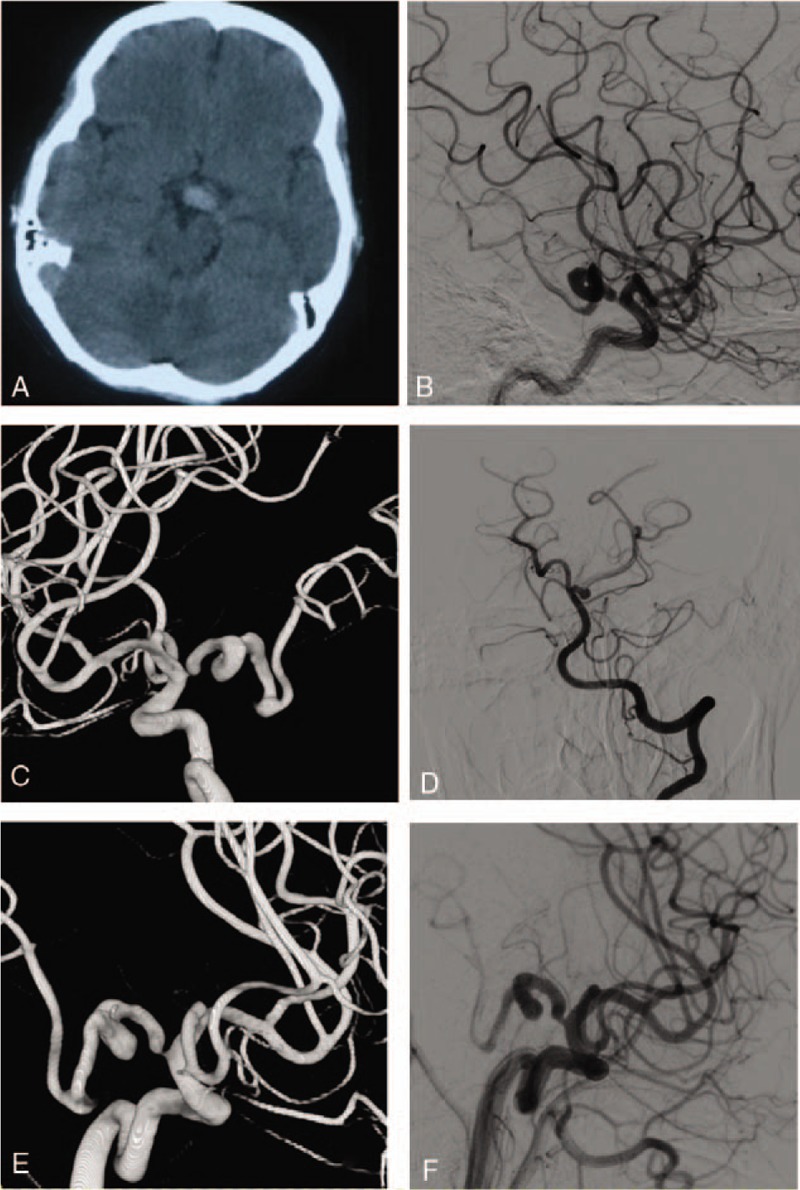
Images of the patient 1. A: Head CT showing high density shade in front of mesencephalon; B: Left ICA injection angiograms showing Pure arterial malformation of LPCOA and PCA which is moderately dilated and has a loose coil-like loop; C: Left ICA 3D injection angiograms; D: Left VA injection angiograms showing Left PCA P1 segment clear; EF: 6 mo FU.

A 36-year-old female patient presented an intermittent headache. The head CT showed a high-density anterior to the pons and the midbrain. The head DSA revealed the left posterior communicating artery being dilated, overlapping, tortuous, and continuous to the P2 segment of the posterior cerebral artery. While other blood vessels were normal. We treated the patient with pain control and neurotrophic medications. The patient's blood pressure was controlled within the normal range. Follow-up DSA 6 months after discharge did not show any morphological changes of the malformations, and the patient denied recurrence of headaches (Fig. 1).

### 2.2 Case 2

**Figure 2:**
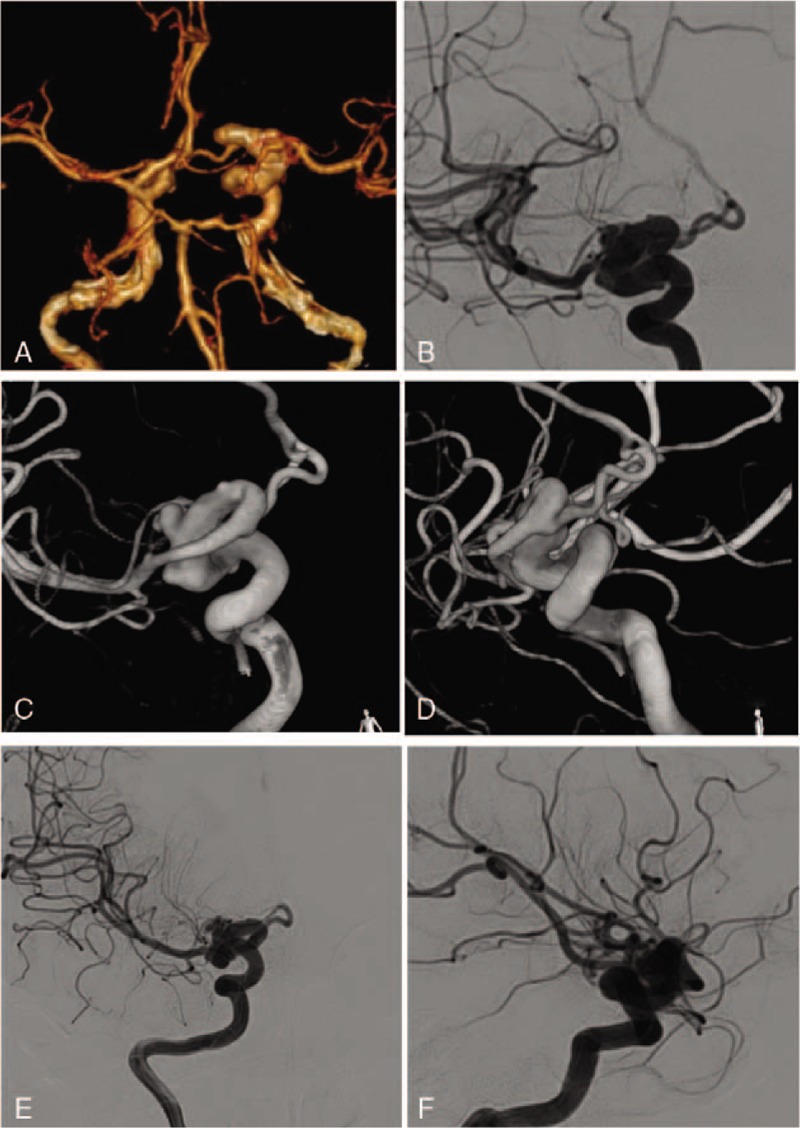
Images of the patient 2. A: Head CTA showing a ectatic and tortuous supraclinoid segment of the Right ICA; B: Right ICA injection angiograms showing a moderately ectatic and loosely coiled supraclinoid segment with 2 superimposed small aneurysms; C: Right ICA 3D injection angiograms; D: Right ICA 3D injection angiograms; EF: 6 mo FU.

A 24-year-old female patient presented to our department with occasional dizziness. The head CTA revealed the dilation of the supraclinoid segment of the right internal carotid artery. Moreover, a local aneurysm-like change was found in the head DSA examination. The patient was given conservative treatment such as neurotrophic drugs and avoiding fatigue, and 6 months later, the head DSA examination showed no changes in the diseased blood vessels. The patient's dizziness symptom was relieved (Fig. 2).

### 2.3 Case 3

**Figure 3:**
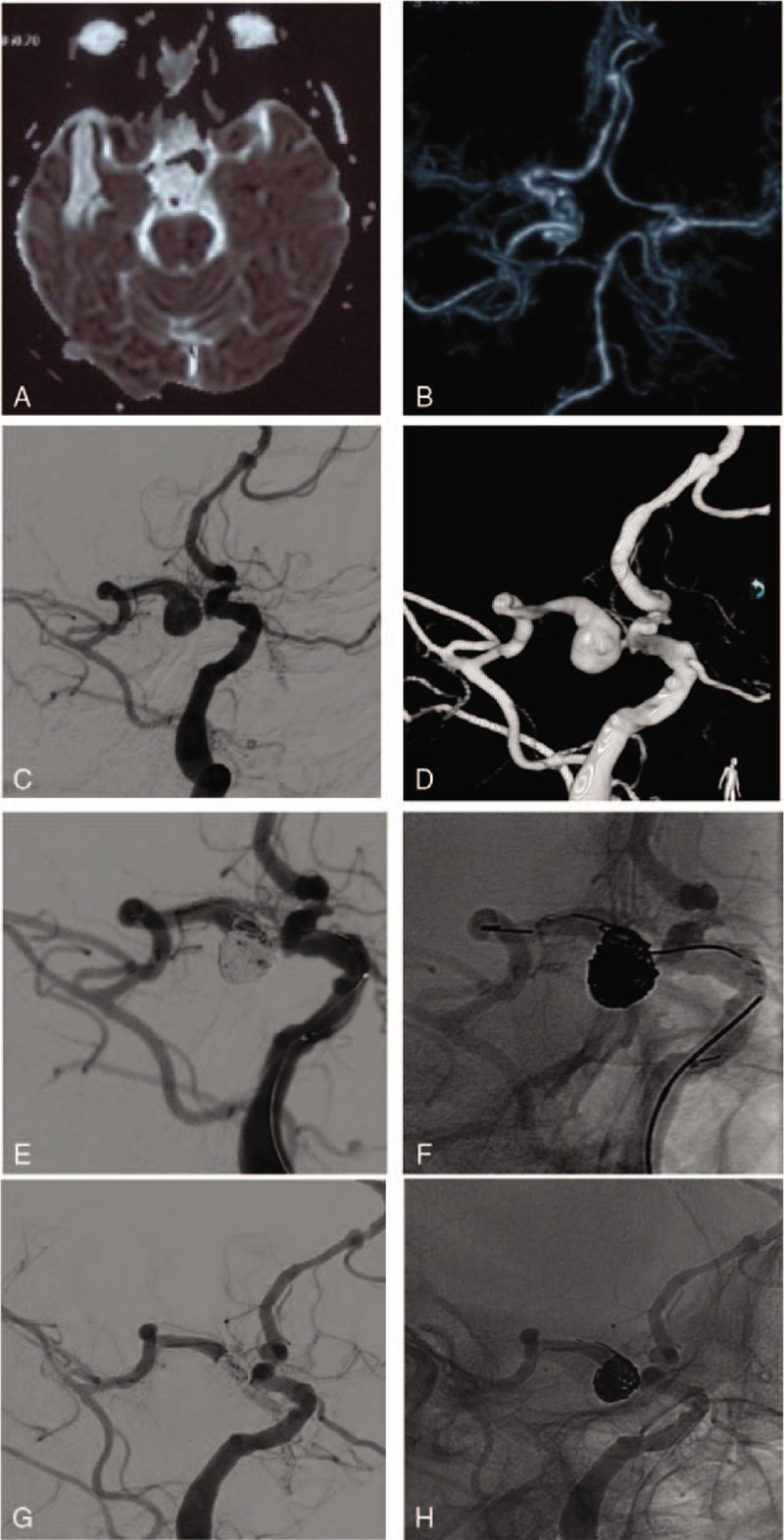
Images of the patient 3. A: Head MRI showing blood flow void beside the brain stem; B: Head CTA showing the ectasia of the left PComA; C: Left ICA injection angiograms showing a ectatic and tortuous PComA and PCA with 1 aneurysm located in PComA; D: Left ICA 3D injection angiograms; E: Embolization of the aneurysm by coils assisted by Stent (silhouette image); F: Embolization of the aneurysm by coils assisted by Stent; G: FU 3 months later, the aneurysm was disappear and the vascular morphology was clear (silhouette image); H: FU 3 mo later, the aneurysm was disappear and the vascular morphology was clear.

A 53-year-old male complained of left limb numbness for 2 weeks. He had a history of hypertension for years and his blood pressure was not controlled well. The head MRI examination showed vascular emptying signals adjacent to the brainstem. The CTA examination revealed an aneurysmal expansion of the left posterior communicating artery. The DSA examination of the head indicated moyamoya disease on the left brain; the proximal portion of the left posterior communicating artery was obviously enlarged, and an aneurysm-like expansion was observed. Moreover, the left posterior communicating artery was distorted, prolonged, and continuous with the posterior cerebral artery. The patient was treated with stent-assisted-coil embolization. Six months later, the patient underwent a DSA review of the head. The vascular lumen remodeling was good, no dilatation was observed, and the aneurysm achieved Raymond I embolization (Fig. 3).

## 3 Ethic statement

Written informed consent was obtained from the patients and their parents for publication of these case reports. Patients have provided informed consent for publication of the cases.

## 4 Discussion

**Figure 4:**
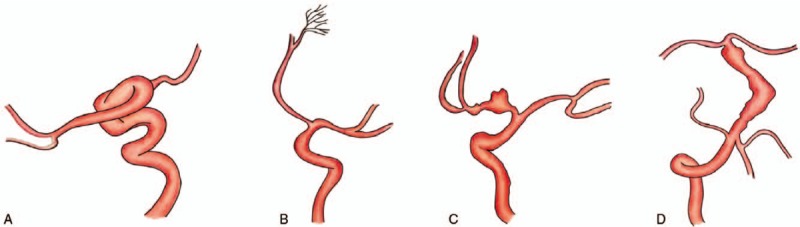
A: Pure arterial malformation(present cases). B: Developmental arterial anomaly or arterial vascular dysplasia. C: Arterial dissection. D: Dilatative arteriopathy or dolichoectasia.

Pure arterial malformation is defined as the presence of dilated, overlapping, and tortuous arteries forming a mass of arterial loops with a coil-like appearance in the absence of any venous components. It may also be associated with local aneurysms or calcification.^[1,3–26]^ The diagnosis of the disease requires DSA and 3D imaging examinations. Pure arterial malformation needs to be differentiated from other vascular diseases such as arteriovenous malformation, arterial dissection, dilatation and prolongation syndrome, and developmental arterial anomalies^[1,11,25–28]^ (Fig. 4). The abnormal arteries neither communicate with veins through the vascular network or vascular nest nor communicate with veins directly. This lack of communication with venous structures is the major characteristic differentiating from arteriovenous malformations and arteriovenous fistulas. Pure arterial malformation can involve any intracranial blood vessels, but it mostly occurs at the supraclinoid segment of the internal carotid artery, the posterior communicating artery, the anterior cerebral artery, and the M1 segment of the middle cerebral artery. The natural history of patients with pure arterial malformation is usually non-specific. On the other hand, the dilated arterial disease occurs mostly in the internal carotid and vertebral basilar artery.^[6,29]^ Most patients with dilated arterial disease also have other risk factors, such as hypertension-related diseases, infectious diseases, immune diseases, or systemic macrovascular dilatation changes.^[10,27,28,30–32]^ Developmental arterial anomalies often involve the distal branches of the intracranial vessels, and they are mainly characterized by small network-like dilated arterial clusters. Developmental arterial anomalies are also associated with embryonic dysplasia, and there may be cerebral cortical dysplasia around them.^[5,6,23]^ In patients with pure arterial malformation, the abnormal vessel wall remains parallel, and the affected vessel segments are longer.^[33]^ The form of the blood vessels and the vascular orientation can be seen clearly. The major feature differentiating pure arterial malformation from arterial dissection is that there is no hematoma in the vascular wall or double cavity sign on imaging examinations. However, theory exists pertaining to the relationship between arterial dissection and pure arterial malformation. Some scholars believe that repetitive healing could take place during the pathological process of arterial dissection by the formation of fusiform expansion, local stenosis, and complex odd shape aneurysms. Therefore, pure arterial malformations is a chronic healing of arterial dissection.^[1,11,25,26,34]^ The etiology and pathogenesis of pure arterial malformation remain unclear. It may occur when there is a defect or injury on the vessel wall because of congenital dysplasia, they may also occur after acquired damage caused by bacterial or viral infection, or they may be a chronic healing process for dissection lesions.^[14]^

The optimal therapy for pure arterial malformation also remains controversial. A review of previous literatures showed that 94.4% of affected patients received conservative treatment and achieved a good prognosis. So far, only 2 cases treated with surgery or endovascular treatment have been reported. Brinzikji et al suggested that patients with pure arterial malformation should be treated conservatively. We proposed that conservative treatment is appropriate for the patients with a pure arterial malformation without a local aneurysm; however, patients with pure arterial malformation with local aneurysms can achieve a good prognosis when treated via intervention with endovascular treatment.^[35,36]^ In the 3 cases reported here, the first 2 patients were young female patients with a good natural history. One of these patients had a left posterior communicating artery malformation, and the other patient had a pure arterial malformation in the supraclinoid segment of the right internal carotid artery with a local aneurysm (the shape of the aneurysm was regular). Considering that the patient's main symptoms were not related to the diseased blood vessels and the patient was young, adopting endovascular treatment or surgical bypass therapy would result in a larger risk and may cause some problems of taking medicines post operation. Therefore, the patient was given conservative treatment. Moreover, the patient was regularly reviewed by imaging examination, and the risk factors for the disease were controlled. After 6 months, a review of a head DSA examination revealed no changes in the diseased blood vessels. The third patient had a history of hypertension for many years. Considering the distorted, dilated PCoA-PCA observed in this patient exhibited aneurysm-like changes, there was a risk of rupture and bleeding. Moreover, the patient had an embryonic posterior cerebral artery. The blood therefore experienced turbulence in the posterior communicating artery of the aneurysm-like expansion, which is a risk factor for thrombosis. Once the thrombus falls off, it may cause infarction in the area which the posterior cerebral artery blood supplies, and may lead to serious consequences. Therefore, the patient was treated with stent-assisted-coil embolization. The stent was placed in the distorted and dilated posterior communicating artery. At the same time, we treated the local aneurysm with coils to reduce the risk of bleeding and remodel the vascular lumen. The patient was given antiplatelet therapy after the operation. After 6 months, a head DSA examination showed that the aneurysm-like dilation had disappeared and the lumen remodeling was good.

We reviewed some individual and series of case reports of pure arterial malformations or similar pure arterial malformations reported in the past (Table 1). Among the 36 reviewed cases, 69.4% were female and 66.7% were young patients aged between 14 and 44 years old. The disease was usually observed in young women.^[14]^ The clinical symptoms varied widely, with 38.9% of the patients presenting with headache, 13.9% with seizures, 8.3% with PHACE, and 8.3% diagnosed by chance, and 30.6% presented with dizziness and hemiplegia, viral infections or other different symptoms. Children may be accompanied by congenital vascular dysplasia or systemic diseases, such as genetic diseases and infections, and they will therefore exhibit other symptoms at presentation.^[6,29–31,37]^ Most elderly patients are associated with hypertension, diabetes, hyperlipidemia and other risk factors for damage to vessel walls. This disease can involve any part of any blood vessel in the brain, with 33% of patients having symptoms suggesting the simultaneous involvement of multiple blood vessels in the brain. Among the cases reviewed, the PCoA-PCA, ICA supraclinoid segment, and MCA-M1 were involved in 64.2%. A total of 6 patients with pure arterial malformations were accompanied with local aneurysm. The lesions which involved PCoA-PCA or MCA were more likely to have local aneurysms or calcification.^[4,10,11,26]^ However, lesions involving the distal end of the ACA were mainly characterized by an expansion in the vascular diameter and was sometimes accompanied by peripheral cerebral cortical dysplasia.^[13,17,19,24]^

**Table 1:**
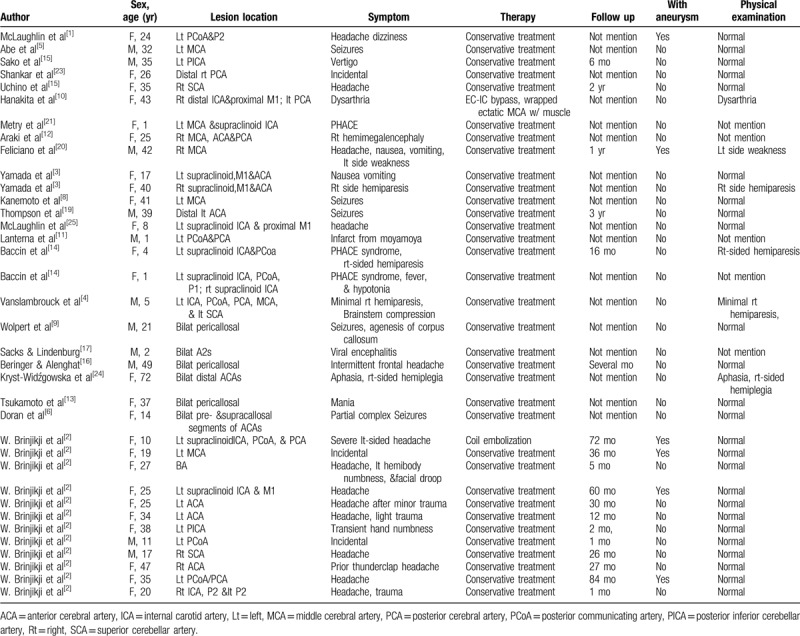
Literature review of patients with pure arterial malformations or similarly pure arterial malformations.

## 5 Conclusion

Among cerebrovascular diseases, pure arterial malformation is a relatively rare disease. Pure arterial malformation is the presence of dilated, overlapping, and tortuous arteries forming a mass of arterial loops with a coil-like appearance in the absence of any venous components. With the increasing awareness of pure arterial malformations, it is beneficial to give the patients conservative treatment, regular imaging examinations and to control risk factors. However, pure arterial malformations with local aneurysm may require surgery or endovascular treatment. The diagnosis and treatment of this disease remains a hot topic of discussion and are worth continued exploration.

## Author contributions

**Conceptualization:** Hong Lei Wang, Ning Xu.

**Data collection and writing of the original article:** Tianyi Liu.

**Data curation:** Hao Meng, Zheng Wan, Jian-Jun Xu, Tianyi Liu

**Development of the idea and editing of the draft:** Hao Meng.

**Drafting of the original article:** Ning Xu.

**Project administration:** Tianyi Liu, Hao Meng.

**Resources:** Tian-Yi Liu, Zhe-Ming Zhang.

**Surgeon performed the operation:** Honglei Wang.

**Writing – original draft:** Zheng Wan, Zheming Zhang, Tian-Yi Liu.

**Writing – review & editing:** Tian-Yi Liu.
